# Is exposure to tobacco associated with extrahepatic cholangiocarcinoma epidemics? A retrospective proportional mortality study in China

**DOI:** 10.1186/s12885-019-5484-9

**Published:** 2019-04-11

**Authors:** Lei Hou, Jingmei Jiang, Boqi Liu, Wei Han, Yanping Wu, Xiaonong Zou, Fang Xue, Yuanli Chen, Biao Zhang, Haiyu Pang, Yuyan Wang, Zixing Wang, Yaoda Hu, Junyao Li

**Affiliations:** 1 Department of Epidemiology and Biostatistics, Institute of Basic Medical Sciences Chinese Academy of Medical Sciences / School of Basic Medicine Peking Union Medical College, A505 Room Mingri Building, 5 Dongdansantiao Street, Dongcheng District, Beijing, 100005 China; 2 Cancer Institute & Hospital, Chinese Academy of Medical Sciences / Peking Union Medical College, Beijing, China; 3 National Center for Chronic and Noncommunicable Disease Control and Prevention, Chinese Center for Disease Control and Prevention, Beijing, China

**Keywords:** Smoking, Cholangiocarcinoma, Mortality, Population

## Abstract

**Background:**

Extrahepatic cholangiocarcinoma (ECC) has become one of the most rapidly increasing malignancies in China during recent decades. The relationship between tobacco exposure and ECC epidemics is unclear; this study aimed to explore this relationship.

**Methods:**

We included 55,806 participants aged 30 years or older from the National Mortality and Smoking Survey of China. Smoking in participants and spouses was defined as 1 cigarette or more per day for up to 1 year. Spouses’ smoking was taken as a measure of exposure to passive smoking. Smoking information in 1980 was ascertained and outcomes were defined as ECC mortality during 1986–1988.

**Results:**

We found that either passive or active smoking increased the risk of death from ECC by 20% (risk ratio [RR], 1.20; 95% confidence interval [CI], 0.99–1.47), compared with no exposure to any tobacco. This risk was a notable 98% (RR, 1.98; 95% CI, 1.49–2.64) for individuals exposed to passive plus active smoking. These findings were highly consistent among men and women. Pathology-based analyses showed dose-response relationships of ECC with pack-years for all types of smoking exposure (*P*s for trend < 0.05); the RR reached 2.75 (95% CI, 1.20–6.30) in individuals exposed to combined smoking with the highest exposure dose. The findings were similar for non-pathology-based analysis.

**Conclusions:**

This study indicates that tobacco exposure increases ECC risk. Given the dramatic increase of exposure to secondhand smoke and patients with ECC, an inadequate provision of smoke-free environments could be contributing to ECC epidemics and could further challenge public health and medical services, based on the current disease spectrum.

**Electronic supplementary material:**

The online version of this article (10.1186/s12885-019-5484-9) contains supplementary material, which is available to authorized users.

## Background

High incidence rates of cholangiocarcinoma (CC) (> 6 per 100,000 people) have been reported in countries of Eastern Asia, such as in China, though this cancer is relatively rare worldwide [1]. CC accounts for 10–20% of deaths owing to hepatobiliary malignancies, which are the second most common cause of death from cancer in China [2, 3]. Given its resistance to current treatments and poor prognosis, the mortality and incidence of CC are virtually the same. From an anatomical viewpoint, CCs are classified as intrahepatic (ICC) or extrahepatic (ECC). ECC has been documented to account for about 90% of CCs [4]. In the Americas, 65% of ECC cases occur in the seventh decade of life [5]. By contrast, our data suggest that there are many middle-aged people with ECC cases among the Chinese population. Compared with a rising trend in ICC, the incidence of ECC seems to be decreasing or remaining constant in most developed countries, such as the United Kingdom and the United States (US) [6, 7]. However, for reasons that are unclear, the ECC appears to be one of the most rapidly increasing cancers in China, in areas such as urban Shanghai [8, 9].

Most cases of ECC are considered de novo with no apparent cause, although several risk factors of CC have been established, such as parasitic infection and hepatolithiasis especially associated with ICC and primary sclerosing cholangitis mainly linked with perihilar CC [3–5]. A recent experimental study indicates that nicotine, a major active component of cigarette smoke, contributes to biliary fibrosis by activation of cholangiocyte proliferation and expression of profibrotic genes [10]. Tobacco smoking is associated with the reduction of p53, a tumor suppressor gene [11], and suppression of T-cell responses by nicotine; in addition, tar is associated with decreased immune surveillance of tumor cells [12]. The experiences of developed countries indicate that tobacco control accounts for nearly all improvements in mortality rates for smoking-related malignancies during the past four decades. However, smoking is currently not an established risk factor of ECC [13]. Despite China having the largest population exposed to tobacco worldwide and a rapidly rising number of ECC cases [14, 15], studies did not indicate an association of smoking with ECC in this population. With a recent systematic review [16], we re-pooled the odds ratios (ORs) of four case-control studies conducted among the Chinese population and still found a negative association between smoking and ECCs (*I*^*2*^ = 3.0%, *P* for *I*^*2*^ = 0.377; OR, 1.00; 95% confidence interval [CI], 0.78–1.22); hospital-based design and a small sample size could contribute to this negative finding. Research conducted on the association between exposure to passive smoking and ECC is lacking worldwide. In the absence of cohort studies, results from case-control studies have provided little evidence on active smoking. Two population-based case-control studies from North America indicated an association between active smoking and ECC [17, 18]; however, other studies from the Netherlands, Italy, and the US did not support this association [19–23].

In any case, smoking is suspected to contribute to this rapidly rising cancer [8]. The aim of the current study is to explore the relationship of tobacco exposure with ECC epidemics by investigating the association between passive or active cigarette smoking, or both, and ECC mortality in Chinese people.

## Methods

### Study population

The China Nationwide Retrospective Mortality Survey included 1,136,686 all-cause deaths among participants aged 30 years or older during 1986–1988, in 24 cities that were the largest in their provinces and 79 rural counties randomly chosen from over 2000 counties in China. These 103 areas covered all geographic and economic zones across 28 provincial administrative regions of China. We identified deaths primarily using local administrative records and medical records. The underlying cause of each death was coded according to the International Classification of Diseases, 9th Revision (ICD-9). Over 500 trained interviewers usually worked as teams of two in urban areas and teams of four in rural areas. Detailed information on this survey has been described elsewhere [24–29].

**Fig. 1:**
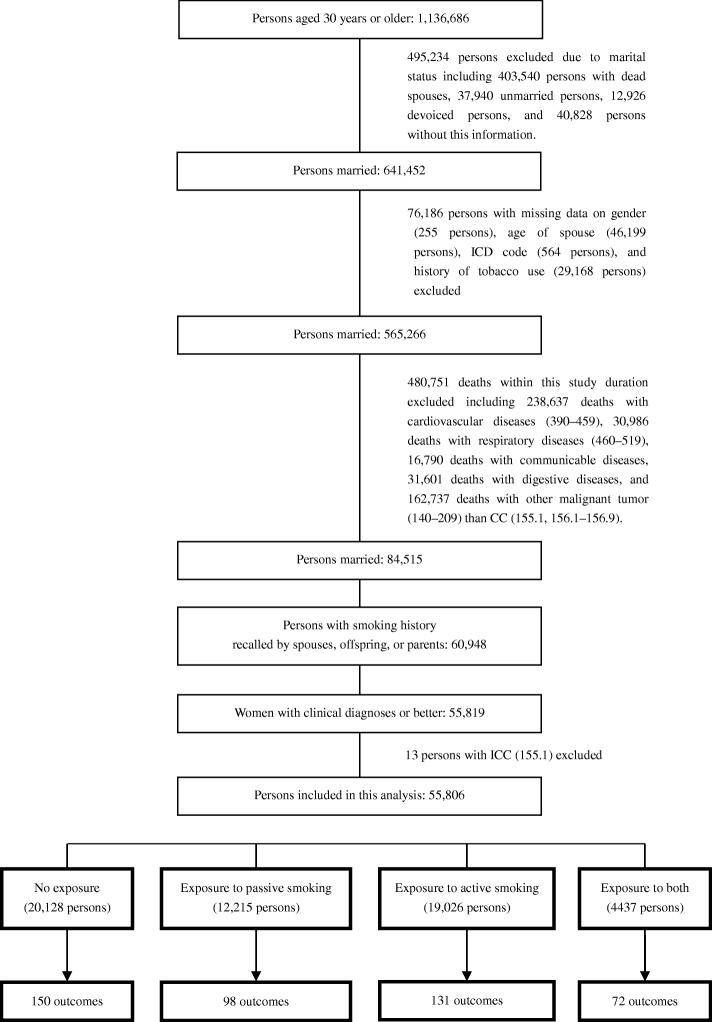
Flow chart for recruitment and grouping of 55,818 participants. Information on exposure to tobacco in 1980 was retrospectively collected from the China Nationwide Retrospective Mortality Survey for deaths during 1986–1988

We included 565,266 married people who had no missing data on gender, age of spouse, and history of tobacco use, to establish this longitudinal-like observed population. We excluded 269,623 participants that died from cardiovascular and respiratory diseases, which are closely associated to tobacco exposure and likely to affect exposure measurement owing to behavior changes after the occurrence of these diseases. In addition, to ensure an initially CC-free status at baseline, we excluded 211,128 participants that died from communicable diseases, digestive diseases, and malignant tumors other than CC. In a comparison between other excluded participants with the remaining participants (Additional file 1: Table S1), we found that excluded individuals with other diseases—who had the most missing data—had a relatively high rate of exposure to both active and passive smoking. Moreover, to avoid bias owing to recall and diagnostic misclassification, we retained only those participants with a smoking history—as recalled by living spouses (74.0%), children (24.2%), or parents (1.8%) who had usually lived with the deceased person for a long time—and those with clinical diagnoses or higher. Finally, after excluding 13 ICC cases, a total of 55,806 participants were included in this retrospective proportional mortality study. Details of enrollment of the study population are shown in Fig. 1.

### Definitions of exposure and grouping

Spouses or other relatives of all deceased persons were interviewed, to obtain information on participants’ smoking history during this mortality survey. Interviewees retrospectively described the smoking habits of the deceased participants, as well as their own, in the year 1980; this guaranteed to minimize effects of behavior changes after the participant was diagnosed with ECC. Smoking in participants and spouses was defined as ≥1 cigarette per day for up to 1 year. Spouses’ smoking was taken as a measure of exposure to passive smoking among participants. To define exposure-years of passive smoking, we used 20 years as the cut-off age for beginning smoking because we had no data on the participants’ date of marriage. Age 20 years is an acceptable and likely cut-off because this was a common age for marriage before the foundation of the People’s Republic of China in 1949 [30]. For spouses who began to smoke at age 20 years or older, we defined years of passive smoking as the age at death for cases or controls minus the age at onset of daily smoking. For spouses who began to smoke before age 20 years, the number of years of smoking before this age was further subtracted [27]. Accordingly, these participants were divided into control and exposure groups (20,128 vs. 35,678 participants), according to smoking information in 1980. The control group was defined as participants with no exposure to tobacco, including passive smoking and active smoking. Individuals exposed to any tobacco use were defined as the exposure group, including nonsmokers exposed to passive smoking (12,215 participants, 21.9% in total), smokers unexposed to passive smoking (19,026 participants, 34.1%), and smokers exposed to passive smoking (4437 participants, 7.9%).

### Ascertainment of ECC

Outcomes were defined as ECC deaths during 1986–1988 after exposure to tobacco (prior to 1980). In this national mortality survey, we ascertained 451 cases of ECC (ICD-9 code: 156.1–156.9), accounting for 97.2% of all CC. These ECC cases were confirmed by autopsy, histological examination, surgical procedure, clinical assessment including imaging and laboratory testing, and clinical manifestations (0.7, 29.7, 12.2, 54.3, and 3.1% of total cases, respectively). Pathology-based diagnoses—including autopsy, histological examination, and surgical procedure which are authorized as the highest category of diagnosis in the national death surveillance system of China—were performed with 42.6%. 50.3, 32.4, and 12.9% of diagnoses were determined at provincial-, prefectural-, and county-level hospitals, respectively. Among 451 deaths owing to ECC, 24 patients have other digestive diseases including 19 related to the gallbladder and biliary tract (ICD-9 codes: 574–576, 188), 3 related to the liver (ICD-9 codes: 571, 573), and 2 related to the gastrointestinal tract (ICD-9 codes: 538, 560). A total 150, 98, 131, and 72 of the 451 deaths occurred in the control group and the three exposure groups listed above.

### Statistical methods

Mean ± standard deviation and person-number (percentage in total) were used for presenting continuous and categorical variables, respectively. We calculated risk ratios (RRs) with 95% confidence intervals (CIs) comparing smokers and nonsmokers. RRs were estimated using unconditional logistic regression models [24, 25]. The initial models (Model 1) were adjusted for age as well as gender if applicable. The fully adjusted models (Model 2) further included variables that could affect association with both exposure and outcomes, including age, gender, urban or rural residence, ethnicity, and education; if applicable, the model also included either passive or active smoking and presence or absence of pathology-based diagnoses. In the interest of investigating a dose-response relationship, we defined 1 pack-year as 20 cigarettes smoked per day for 1 year. For smokers exposed to passive smoking, pack-years of passive plus active smoking were defined as active smoking multiplied by passive smoking; this was owing to the likely weak effect of each single variable on outcomes, including passive or active smoking, years of exposure, and cigarettes smoked per day.

Sensitivity analysis was used to assess the robustness of validity. First, ECC-free status at baseline was considered. The 5-year survival rate for ECC was approximately 10% during 1979–2004 in the US [31]; this rate was likely lower in China. In other words, among all ECC deaths in our survey, there were very few participants with ECC (e.g., < 10%) in 1980, when their smoking status was affirmed. Accordingly, 10% of deaths owing to ECC in each subgroup by gender and 10-year age span were randomly deleted from the participant database and a reanalysis was carried out. In addition, non-pathology-based diagnoses or digestive diseases other than ECC are factors that likely lead to misclassification of outcomes; therefore, in a random stepwise manner, we changed 10, 20, 30%, …, 100% of these ECC deaths to non-outcomes and recalculated the effect values. Lastly, to elaborate the variable of exposure to passive smoking, we showed the results considering the assumed age of marriage.

All analyses were performed using SAS 9.2 statistical software (SAS Institute, Inc., Cary, NC, USA). All *P*-values were two sided except *P* trend tests based on unconditional logistic regression, in which one-sided *P* values were used. A *P*-value < 0.05 was considered statistically significant.

## Results

Similar characteristics of gender, age, residence, ethnicity, and education level were observed between controls and participants exposed to tobacco. We also found large differences in these characteristics between the three exposure groups (Table 1); however, these participants with exposure to tobacco had median exposure time of 30 years or over and a median daily exposure of 10 cigarettes, for both passive and active smoking; this resulted in a generally long pack-year history in this population.

**Table 1:** Characteristics of control and tobacco-exposure groups

	Controls	Exposure to tobacco				Total
		Passive	Active	Passive & Active	All	
Men (%)	59.3	9.2	95.5	63.6	62.0	61.0
Age (years)	57.9 ± 15.7	53.4 ± 14.5	56.9 ± 14.9	63.5 ± 11.2	56.6 ± 14.7	57.0 ± 15.1
Urban residence (%)	75.8	70.7	72.3	86.3	73.5	74.3
Han nationality (%)	95.4	96.6	95.6	97.8	96.2	95.9
Education (%)						
College or university	5.5	1.6	3.2	2.1	2.5	3.6
Middle school	26.0	20.1	28.4	16.6	24.1	24.8
Primary school	35.1	32.9	42.1	41.8	38.9	37.5
Illiteracy	31.7	43.9	25.1	38.4	33.2	32.6
Unknown	1.8	1.6	1.3	1.2	1.4	1.5
Years exposed to passive smoking	–	30 (18–40)	–	36 (26–44)	–	–
Cigarettes passively smoked daily	–	10 (6–20)	–	10 (4–17)	–	–
Pack-years exposed to passive smoking	–	15 (6–27)	–	14 (4–27)	–	–
Years exposed to active smoking	–	–	34 (21–46)	40 (30–49)	–	–
Cigarettes actively smoked daily	–	–	10 (6–20)	10 (5–20)	–	–
Pack-years exposed to active smoking	–	–	17 (7–32)	19 (6–35)	–	–

The fully-adjusted model showed that exposure to tobacco increased the risk of death from ECC by 20% with marginal statistical significance (RR, 1.20; 95% CI, 0.99–1.47), as compared with non-exposure. However, this risk was much different when comparing the three exposure subgroups to controls. RRs were 1.15 (0.86–1.55) and 1.11 (0.85–1.44) for nonsmokers exposed to passive smoking and for smokers unexposed to passive smoking, respectively; for smokers exposed to passive smoking, the RR increased dramatically to 1.98 (1.49–2.64). These findings were highly consistent among both male and female participants as well as between pathology-based and non-pathology-based analyses (Table 2). Among participants with ECCs, 2.4, 8.9, 28.4, 37.7, 20.8, and 1.8% were in the age of 30–39, 40–49, …, 70–79, and 80 years or older, respectively. RRs for an association between exposure to passive plus active smoking and ECC death were strongly influenced by age, and both crude and adjusted RRs began increasing at age 50 years.

**Table 2:** Gender-, age-, and diagnosis-specific relationship between exposure to tobacco and death owing to extrahepatic cholangiocarcinoma

	Numbers of outcomes	Numbers of persons observed	‰	RR (95% CI)	
				Model 1	Model 2
Men					
Control	86	11,937	7.2	1.00	1.00
Passive	14	1122	12.5	1.61 (0.91–1.85)	1.56 (0.88–2.79)
Active	115	18,162	6.3	0.93 (0.70–1.23)	0.97 (0.73–1.29)
Passive & Active	39	2820	13.8	1.81 (1.23–2.66)	1.67 (1.14–2.46)
Women					
Control	64	8191	7.8	1.00	1.00
Passive	84	11,093	7.6	1.08 (0.77–1.50)	1.09 (0.78–1.52)
Active	16	864	18.5	2.15 (1.23–3.74)	1.96 (1.16–3.43)
Passive & Active	33	1617	20.4	2.46 (1.61–3.76)	2.56 (1.66–3.94)
30~49 years					
Control	21	6320	3.3	1.00	1.00
Passive	17	4971	3.4	1.31 (0.58–2.99)	1.49 (0.63–3.43)
Active	11	6091	1.8	0.52 (0.23–1.16)	0.56 (0.25–1.25)
Passive & Active	2	456	4.4	0.91 (0.21–3.96)	0.98 (0.22–4.27)
50~69 years					
Control	89	8457	10.5	1.00	1.00
Passive	67	5333	12.6	1.07 (0.75–1.54)	1.07 (0.75–1.54)
Active	94	8551	11.0	1.23 (0.88–1.71)	1.25 (0.90–1.75)
Passive & Active	48	2587	18.6	1.75 (1.23–2.49)	1.72 (1.20–2.46)
70~98 years					
Control	40	5351	7.5	1.00	1.00
Passive	14	1911	7.3	1.08 (0.56–2.10)	1.09 (0.56–2.11)
Active	26	4384	5.9	0.76 (0.45–1.29)	0.77 (0.45–1.30)
Passive & Active	22	1394	15.8	1.96 (1.16–3.33)	1.84 (1.08–3.13)
Pathology-based					
Control	54	4501	12.0	1.00	1.00
Passive	45	2971	15.1	1.35 (0.83–2.19)	1.41 (0.87–2.28)
Active	62	5537	11.2	1.25 (0.82–1.90)	1.26 (0.89–2.07)
Passive & Active	31	836	37.1	2.50 (1.59–3.93)	2.38 (1.50–3.77)
Non-pathology-based					
Control	96	15,627	6.1	1.00	1.00
Passive	53	9244	5.7	0.98 (0.67–1.43)	1.00 (0.69–1.46)
Active	69	13,489	5.1	0.91 (0.65–1.28)	0.96 (0.68–1.35)
Passive & Active	41	3601	11.4	1.79 (1.23–2.58)	1.72 (1.19–2.50)
Total					
Control	150	20,128	7.5	1.00	1.00
Passive	98	12,215	8.0	1.15 (0.86–1.53)	1.15 (0.86–1.55)
Active	131	19,026	6.9	1.08 (0.83–1.41)	1.11 (0.85–1.44)
Passive & active	72	4437	16.2	2.06 (1.55–2.73)	1.98 (1.49–2.64)

**Fig. 2:**
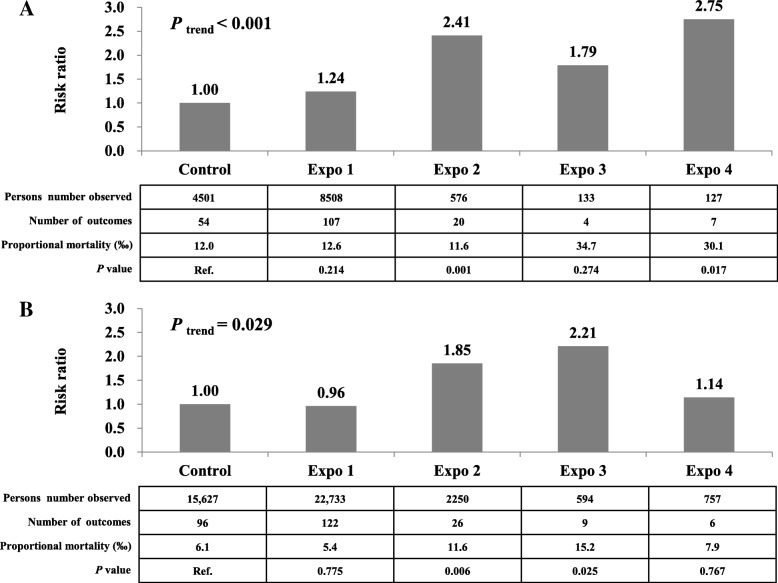
Dose-response relationship of exposure to tobacco with deaths owing to extrahepatic cholangiocarcinoma. **a** Pathology-based analysis; **b** Non-pathology-based analysis Control defined as no exposure to any tobacco use; Expo 1 defined as exposure to either passive or active smoking only; Expo 2 to 4 defined as combined exposure to both passive and active smoking with fewer than 400, 400–799, and 800 pack-years squared or more, respectively. Risk ratios adjusted for age, gender, urban or rural residence, ethnicity, and education (Model 2)

We used pack-years to demonstrate a dose-response relationship owing to a weak effect of single variables for tobacco exposure on this disease (Table 3 and Fig. 2). In the pathology-based analysis, the highest dose of pack-years (≥ 20) increased the risk of ECC mortality by 60% (RR, 1.60; 95% CI, 0.94–2.75) for passive smoking (*P* for trend = 0.021) and 66% (RR, 1.66; 95% CI, 1.03–2.67) for active smoking (*P* for trend = 0.045), compared with controls. We further combined non-smokers exposed to passive smoking and smokers unexposed to passive smoking as a single exposure group and divided smokers exposed to passive smoking into three exposure subgroups with stepwise increasing doses, i.e., less than 400, 400–799, and 800 pack-years squared or more. The risk of ECC death significantly increased with increasing exposure dose (*P* for trend < 0.001). This risk dramatically increased to 2.75 fold (RR, 2.75; 95% CI, 1.20–6.30) in participants with the highest exposure dose, compared with controls. These findings were highly consistent in the non-pathology-based analysis.

**Table 3:** Dose-response relationship between exposure to either passive or active smoking and deaths owing to extrahepatic cholangiocarcinoma

Doses exposed to tobacco	Numbers of outcomes	Numbers of persons observed	‰	RR (95% CI)	
(pack-years)				Model 1	Model 2
Pathology-based					
Control	54	4501	12.0	1.00	1.00
Exposure to passive smoking					
1~9	81	6976	11.6	1.13 (0.66–1.96)	0.94 (0.46–1.95)
10~19	17	1105	15.4	1.62 (0.90–2.91)	1.09 (0.51–1.36)
≥ 20	40	1267	31.6	1.97 (1.24–3.15)	1.60 (0.94–2.75)
*P* for trend				< 0.001	0.021
Exposure to active smoking					
1~9	70	5271	13.3	1.12 (0.68–1.85)	0.86 (0.45–1.63)
10~19	20	1646	12.2	1.59 (0.91–2.75)	1.26 (0.65–1.41)
≥ 20	48	2428	19.8	1.78 (1.15–2.76)	1.66 (1.03–2.67)
*P* for trend				0.002	0.045
Non-pathology-based					
Control	96	15,627	6.1	1.00	1.00
Exposure to passive smoking					
1~9	95	17,933	5.3	1.04 (0.67–1.63)	0.79 (0.42–1.48)
10~19	16	3276	4.9	0.86 (0.50–1.49)	0.79 (0.41–1.52)
≥ 20	52	5133	10.1	1.61 (1.12–2.32)	1.27 (0.81–1.98)
*P* for trend				0.013	0.220
Exposure to active smoking					
1~9	83	14,506	5.7	1.08 (0.70–1.64)	0.73 (0.40–1.34)
10~19	18	3498	5.1	0.92 (0.55–1.56)	0.95 (0.53–1.70)
≥ 20	62	8330	7.4	1.24 (0.88–1.76)	1.08 (0.73–1.60)
*P* for trend				0.066	0.431

A sensitivity analysis showed that our results were robust. After randomly deleting 10% of deaths owing to ECC were randomly deleted from the participant group, compared with non-exposure, the risk of death from ECC increased by 9% (RR, 1.09; 95% CI, 0.80–1.50), 20% (RR, 1.20; 95% CI, 0.91–1.59), 113% (RR, 2.13; 95% CI, 1.58–2.87), and 23% (RR, 1.23; 95% CI, 1.00–1.52) for the three exposure groups and all exposed participants, respectively. After 269 cases with non-pathology-based diagnoses or digestive diseases other than ECC were changed to non-outcomes, the risks increased by 34% (RR, 1.34; 95% CI, 0.81–2.21), 38% (RR, 1.38; 95% CI, 0.90–2.12), 152% (RR, 2.52; 95% CI, 1.58–4.00), and 39% (RR, 1.39; 95% CI, 1.00–1.93), respectively. The findings retained robust with 10–90% of these cases changed (Table 4). In addition, the assessment of smoking in either spouse or parents/children did not show likelihood of recall bias; the RRs for all exposed participants were 1.21 (95% CI, 0.96–1.52) and 1.19 (95% CI, 0.81–1.75), respectively. Moreover, owing to age of marriage, some participants with spouses who smoked would not actually be exposed to passive smoking. Additional file 1: Table S2 shows RRs under the assumed age of marriage 20 years old. When changing this to age 25 or 30 years, the results were retained.

**Table 4:** Sensitivity analysis for 269 participants with non-pathology-based diagnoses or digestive diseases other than cholangiocarcinoma

% changed to non-cases	Number of cases changed	RR (95% CI)* for exposure to tobacco			
		Passive	Active	Passive & Active	All
100	269	1.34 (0.81–2.21)	1.38 (0.90–2.12)	2.52 (1.58–4.00)	1.39 (1.00–1.93)
90	243	1.26 (0.79–2.00)	1.39 (0.93–2.04)	2.53 (1.65–2.88)	1.40 (1.03–1.90)
80	215	1.39 (0.90–2.14)	1.29 (0.88–1.87)	2.52 (1.70–3.76)	1.41 (1.06–1.88)
70	188	1.43 (0.97–2.10)	0.99 (0.70–1.40)	1.91 (1.30–2.82)	1.18 (0.91–1.54)
60	160	1.51 (1.05–2.18)	0.96 (0.69–1.33)	1.97 (1.37–2.84)	1.21 (0.94–1.55)
50	135	1.42 (1.00–2.03)	1.15 (0.83–1.58)	2.27 (1.61–3.31)	1.33 (1.04–1.70)
40	108	1.14 (0.82–1.59)	1.01 (0.75–1.37)	1.87 (1.34–2.60)	1.13 (0.90–1.41)
30	81	1.16 (0.84–1.60)	1.08 (0.81–1.44)	1.95 (1.42–2.69)	1.18 (0.95–1.47)
20	54	1.19 (0.87–1.62)	1.01 (0.76–1.34)	1.95 (1.44–2.65)	1.17 (0.94–1.44)
10	27	1.20 (0.89–1.63)	1.07 (0.82–1.40)	1.92 (1.42–2.58)	1.21 (0.99–1.49)

*Adjusted for age, gender, urban or rural residence, ethnicity, education, and pathology-based or non-pathology-based diagnosis (Model 2)

## Discussion

This is the first study indicating an association between exposure to tobacco and ECCs in China. Our main findings showed that exposure to tobacco, particularly passive plus active smoking, increased the risk of death owing to ECC with a significant dose-response relationship.

Our findings are supported by reports on the contribution of cigarette smoke to malignant change in biliary cells [19–21]. However, our study differs from previous observational studies that have discussed the effect of active smoking but have lacked information about passive smoking. Our controls had no exposure to either passive or active smoking, based on information of passive smoking obtained from family members; therefore, we could simultaneously observe the effects of passive, active, and passive plus active smoking on ECC. Our study highlighted that a high interaction between passive smoking and active smoking could contribute to ECC mortality, as suggested by other studies on the association between smoking and cancers such as cervical cancer [28]. Based on our control design and a large number of participants with long-term heavy exposure to tobacco in our database (Table 1), we could further define multiple doses of exposure to passive plus active smoking by combining information on both passive and active smoking with years of smoking and cigarettes smoked daily. Using pack-years squared exposed, we found a 2.75-fold increased risk of ECC mortality in the exposure group with the highest dose, as compared with controls.

Our study is especially practical for the prevention and control of this rapidly increasing fatal disease in the Chinese population. China has 300 million smokers and a relatively stable pattern of active smoking, reflected in the prevalence rates and cigarettes smoked daily [29, 32]. However, the exposure rate to secondhand smoke among nonsmokers dramatically increased by 80% during 1984–2010, from 40 to 72.4%, following a change in exposure locations from predominantly in the home to a dramatic increase of smoking in public spaces, including in the workplace [33]. Surveys have also suggested that a growing population of smokers is exposed to passive smoking, and our data included many such individuals. The International Tobacco Control Policy Evaluation China Survey indicated compared with nonsmokers, smokers are more likely to be exposed to secondhand smoke owing to lower levels of about the harm of secondhand smoke [34]. Our study further indicated greater harms owing to combined tobacco exposure in this population than that for nonsmokers exposed to passive smoking. More importantly, high rates of exposure to tobacco are associated with a broad spectrum of diseases. As implied in our study, high rates of exposure to secondhand smoke likely increase the incidence of some rare and refractory cancers, such as ECC. The dramatic increase in such cases poses a challenge to current medical services and public health services, considering that there are much fewer studies on such cancers than those for more common malignancies. We suggest that the age peak for ECC occurrence in China is much earlier than that in the developed countries [9]; our study findings indicate that the association of exposure to tobacco with ECC is particularly prominent among people aged 50 years or older, representing nearly 90% of ECC cases. Such findings could contribute to risk stratification and case detection among patients attending ECC clinics for the diagnosis and treatment of ECC.

A key strength of this research is that despite being a retrospective study, this was a large population-based study across China in which temporality could be established owing to a high fatality rate of this disease and a time interval of more than 5 years between exposure and outcome. High fatality in this cancer also reduced competing risks from other deaths and risk of exposure change from occurrence of this disease. In addition, we firstly demonstrated dose-relationships between all types of exposure to tobacco and ECC risk as well as interaction between different exposures. Lastly, we used sensitivity analyses to address concerns regarding a retrospective study, such as prevalence of ECC at the beginning time of exposure and misclassification owing to diagnosis; robust results were retained in all analyses.

This study has some limitations. First, although the participants included into this analysis were all from source population, owing to the proportional mortality design and exclusion of some deaths related to smoking, the association between exposure to smoking and ECC could not be established; thus, further evidence is required. Second, the source of passive smoking might be from public areas; however, a low rate of tobacco exposure in public spaces, including workplaces (5.74%), from the 1984 National Smoking Survey of China suggests that study participants had little likelihood of being exposed to tobacco in public spaces. Third, based on the previously low rate of quitting smoking (4.78%) in China, participant behaviors would not be substantially changed between exposure and death owing to ECC. Fourth, we could not completely exclude likely confounding from chronic disorders such as parasitic infections, hepatolithiasis, and liver diseases. However, there was likely to be little bias owing to the exclusion criteria used (although these might have led to selection bias), sensitivity analysis conducted, and little established association between these disorders and ECC. Finally, exposure to passive smoking seemed associated with higher risk of ECC than exposure to active smoking in some subgroups; this was related to a smaller sample size after stratification and weak effect of exposure on this disease. Nevertheless, these limitations would not change our main findings. Additionally, our dataset is old, but this did not affect our exploration of this association because this is the only national dataset on this topic that includes spousal information. Moreover, the large sample size could yield sufficient outcomes defined as relatively rare cancers and etiological associations cannot be unchanged over time.

## Conclusions

This study provides the first evidence to support an association of exposure to tobacco with ECC. Given the notably increased ECC risk from passive plus active smoking and a dramatic increase of exposure to secondhand smoke, attention should be given to any change in the tobacco-associated disease spectrum. Tobacco prevention and cessation programs, and provision of smoke-free environments in particular, could contribute to staving off a sharp increase of ECC in China.

## Additional file

Additional file 1: **Table S1.** Characteristics of excluded participants. **Table S2.** Association between exposure and deaths owing to extrahepatic cholangiocarcinoma, considering the assumed age of marriage. (DOCX 16 kb)

## Abbreviations

CC: Cholangiocarcinoma
CI: Confidence interval
ECC: Extrahepatic cholangiocarcinoma
ICC: Intrahepatic cholangiocarcinoma
ICD-9: International Classification of Diseases, 9th Revision
OR: Odds ratio
RR: Risk ratio
