# Supplementary material for: Is exposure to tobacco associated with extrahepatic cholangiocarcinoma epidemics? A retrospective proportional mortality study in China
Source: BMC Cancer. 2019 Apr 11;19:348. doi: 10.1186/s12885-019-5484-9 (PMC6458766; doi:10.1186/s12885-019-5484-9)
Supplement: Supplementary file 1 — Table S1. Characteristics of excluded participants. Table S2. Association between exposure and deaths owing to extrahepatic cholangiocarcinoma, considering the assumed age of marriage. (DOCX 16 kb) [file 12885_2019_5484_MOESM1_ESM.docx]

**Additional files**

**Additional file 1. Characteristics of excluded participants**

|  | Exclusion due to missing data | | | | | Exclusion due to diseases |
| --- | --- | --- | --- | --- | --- | --- |
|  | | Gender | Age of spouse | ICD code | History of tobacco |  |
| N | | 255 | 46,199 | 564 | 29,168 | 480,751 |
| Men (%) | | – | 54.0 | 67.7 | 67.3 | 67.1 |
| Age (years) | | 64.1 ± 12.7 | 67.7 ± 13.4 | 61.4 ± 12.9 | 64.7 ± 11.8 | 63.6 ± 11.8 |
| Exposure to tobacco (%) | |  |  |  |  |  |
| Active only | | 12.2 | 10.6 | 18.8 | – | 16.9 |
| Passive only | | 47.8 | 60.1 | 44.2 | – | 39.2 |
| Both | | 11.8 | 5.6 | 4.4 | – | 11.3 |

**Additional file 2. Association between exposure and deaths owing to extrahepatic cholangiocarcinoma, considering the assumed age of marriage**

|  | Control | Passive | Active | Pasive & Active | All |
| --- | --- | --- | --- | --- | --- |
| Person number at baseline | 20,464 | 11,879 | 19,045 | 4418 | 35,342 |
| Outcome number | 150 | 98 | 131 | 72 | 301 |
| RR (95% CI)^*^ | 1.00 | 1.23 (0.92–1.64) | 1.16 (0.89–1.51) | 2.00 (1.50–2.66) | 1.26 (1.04–1.54) |

^*^Adjusted for age, gender, urban or rural residence, ethnicity, and education (Model 2).
